# Regression Analysis of the Main Environmental Risk Factors for Mountainous Subtype of Zoonotic VL in China

**DOI:** 10.1155/jotm/7495572

**Published:** 2026-09-18

**Authors:** Zhongqiu Li, Haobo Ni, Zhengbin Zhou, Shuxun Wang, Zixin Wei, Yi zhang, Junhu Chen, Shizhu Li

**Affiliations:** ^1^ National Institute of Parasitic Diseases, Chinese Center for Disease Control and Prevention (Chinese Center for Tropical Diseases Research), NHC Key Laboratory of Parasite and Vector Biology, WHO Collaborating Center for Tropical Diseases, National Center for International Research on Tropical Diseases, National Key Laboratory of Intelligent Tracking and Forecasting for Infectious Diseases, Shanghai 200025, China; ^2^ Shanghai Center for Disease Control and Prevention, Shanghai 200025, China; ^3^ School of Global Health, Chinese Center for Tropical Diseases Research, Shanghai Jiao Tong University School of Medicine, Shanghai 200025, China, shsmu.edu.cn

**Keywords:** altitude, logistic regression, mountain-type zoonotic VL, NDVI, temperature, visceral leishmaniasis

## Abstract

**Background:**

Mountain‐type zoonotic visceral leishmaniasis (MT‐ZVL) has re‐emerged in China in recent years, posing a growing public health concern. Canines are the primary reservoir hosts, yet the environmental determinants of canine infection remain insufficiently understood. This study aimed to identify key environmental factors associated with MT‐ZVL infection.

**Methods:**

A total of 1484 canine blood samples were collected from 89 villages across five endemic provinces in China. After excluding cases with missing data, 1374 cases were ultimately included in the analysis. Candidate predictors included canine hair length, age, sex, weight, NDVI, altitude, and temperature. We developed a model using ridge‐penalized logistic regression, tuned hyperparameters, and validated internally through 30 iterations of 10‐fold stratified cross‐validation. Model discriminatory power was evaluated using the area under the receiver operating characteristic (AUC) curve and its 95% confidence interval. Additionally, the optimal cutoff value corresponding to the maximum Youden’s index was determined, and sensitivity, specificity, positive predictive value, negative predictive value, and accuracy were calculated. Variable importance was ranked based on the absolute values of the standardized coefficients.

**Results:**

The final model, built using ridge regression (optimal *λ* = 0.0215), included seven predictors: canine hair length, age, sex, weight, NDVI, altitude, and temperature. Based on 30 repetitions of 10‐fold stratified cross‐validation, the model demonstrated moderate discriminatory power, with an AUC of 0.68 (95% CI 0.62–0.74). The optimal threshold, determined by maximizing the Youden index, was 0.024. At this threshold, sensitivity reached 97.8%, specificity was 35.9%, the negative predictive value was as high as 99.8%, the positive predictive value was 4.9%, and the overall accuracy was 37.9%. Among the variables, NDVI had the highest relative importance (OR = 14.51), followed by canine hair length. This model has an extremely low risk of missed diagnoses and is suitable for screening to rule out the disease; however, due to its high false‐positive rate, it should not be used alone for definitive diagnosis.

**Conclusion:**

Environmental factors, particularly vegetation coverage, play a critical role in shaping the risk of canine infection in MT‐ZVL endemic areas. These findings provide important evidence for improving surveillance strategies and implementing targeted control measures.

## 1. Introduction

Visceral leishmaniasis (VL), also known as kala‐azar, is the second most fatal parasitic disease worldwide after malaria [1]. There are 50,000–90,000 new cases worldwide each year [2]. Without timely treatment, more than 95% of patients will die from complications within 1 to 2 years after the onset of the disease [3]. In 2008, VL was listed as a neglected tropical disease by the World Health Organization (WHO) [4]. Subsequently, a roadmap for the prevention and control of neglected tropical diseases was released by WHO from 2021 to 2030; VL was listed as a public health issue that must be eliminated [5].

In the course of the development of VL in China, three types of VL have been defined based on the epidemiological characteristics, including mountain‐type zoonotic VL (MT‐ZVL), anthroponotic VL (AVL), and desert‐type zoonotic VL (DT‐ZVL) [6, 7]. There were approximately 530,000 VL patients in the early days of the founding of the People’s Republic of China [8]. After effective prevention and control, the prevalence of VL was brought under control in the vast majority of areas across the country in 1958 [7]. However, there were still a few reported cases in the western regions [9]. Since the 21st century, with the changes in the ecological and social environment, VL has generally shown a low prevalence in China. However, the MT‐ZVL has seen a significant resurgence of the epidemic [17].

The MT‐ZVL was prevalent in northern Beijing, Henan, northern Hebei, southern Gansu, northern and southern Shanxi, and northwestern Sichuan [11]. Canines are the definitive host of Leishmania. The MT‐ZVL is transmitted through the bite of sandflies on positive canines [12]. It is impossible that Leishmania spreads vertically through sandflies [13]. When the epidemic season comes each year, newly hatched sandflies do not have Leishmania infection in their bodies. It is only through biting positive canines that sandflies become infectious. Therefore, it is of great significance to know the proportion of positive canines for MT‐ZVL prevention and control. In addition to understanding the probability of canine positivity in this study, it is important to determine the factors contributing to canine infection.

This research involved canine surveys in five endemic provinces across China. It studied the influence of VL‐important reservoir canines’ positive relationship with the main environmental factors (annual average temperature, normalized difference vegetation index [NDVI], and altitude). By establishing and validating a binary logistic regression model, we sought to identify the important reservoir of VL canines’ positive rate and the relationship with environmental factors. These findings provide a reference for formulating more effective, targeted prevention and control measures and for allocating health resources reasonably.

## 2. Materials and Methods

### 2.1. Data Sources and Methods

#### 2.1.1. Canine Data

In this research, canine blood samples were collected from 89 villages located in five provinces (municipalities) in China, including Beijing, Gansu, Henan, Hebei, and Shanxi, all of which are recognized endemic areas for mountain‐type zoonotic visceral leishmaniasis (MT‐ZVL). These provinces were selected because they have reported persistent human MT‐ZVL cases in recent years and represent the major endemic regions of the disease in China. Within each province, villages with a recent history of human VL cases or ongoing canine VL surveillance were prioritized for field investigation. Domestic dogs residing in these villages were recruited during routine epidemiological investigations and surveillance activities conducted in 2023. Blood samples were collected from apparently healthy dogs with the owners’ consent, regardless of breed or sex. A total of 1484 canine blood samples were collected. Among the sampled dogs, 53 breeds were clearly identified, while the breeds of eight dogs could not be determined. Sampling was conducted between May and December 2023, corresponding to the annual field surveillance period in the endemic areas. Among these samples, we excluded 65 cases of weight loss and 53 cases of inability to determine canine hair length, resulting in a total of 110 cases (of which 8 cases had both missing variables); finally, 1374 cases were included in the analysis. At the same time, the basic information of the canines was recorded in the survey. These records contained the latitude and longitude position information while collecting samples. Other state variables of the canines were matched by latitude and longitude information. The Medical Ethics Committee of the National Institute of Parasitic Diseases (NIPD), Chinese Center for Disease Control and Prevention (Chinese Center for Tropical Diseases Research), approved this study (2021019).

#### 2.1.2. Laboratory Test Data

The blood samples of the canines were collected on the spot and placed in a low‐temperature preservation box before being taken back to the laboratory. All blood samples were tested using the Kalazar Detect (Canine) (USA, InBios International, lnc.).

#### 2.1.3. Environmental and Socioeconomic Predictor Variables

Climate and socioeconomic conditions have an important impact on canine infection. For meteorological data, we collected the annual average temperature during the study period. In addition, other predictors (e.g., NDVI, altitude) were collected for our analysis. NDVI represents the health status of vegetation, and its value is between −1 and + 1. The higher the NDVI value, the denser and healthier the vegetation, and vice versa. The spatial resolution of the data is 1 × 1 km. The temperature data are from the National Tibetan Plateau/Third Pole Environment Data Center. NDVI data were obtained from the Terra Moderate Resolution Imaging Spectroradiometer (MODIS) Vegetation Indices Monthly (MOD13A3) and aggregated as annual averages.

#### 2.1.4. Model Construction

The final analysis included a total of 1374 dogs, of which only 45 (3.28%) tested positive. Due to the extremely low frequency of positive cases, the data were not split into training and test sets; instead, all samples were used for modeling and internal validation to avoid evaluation instability caused by an insufficient number of positive cases in the test set. To control for overfitting, an L2 norm penalty was applied to the logistic regression coefficients using ridge regression (*α* = 0). The selection of the regularization strength *λ* is embedded within the cross‐validation process: a 10‐fold hierarchical cross‐validation with 30 repetitions is used, with the area under the ROC curve (AUC) as the optimization objective, to search for the optimal *λ* within a predefined *λ* grid (10^−3^ to 10^0^, select 10 numbers at equal intervals between −3 and 0). The final model is determined by all coefficients corresponding to the optimal *λ*. Based on the average probability from this cross‐validation, the AUC and its DeLong 95% confidence interval were calculated; simultaneously, the optimal classification threshold was determined based on the Youden index (sensitivity + specificity − 1), and the sensitivity, specificity, positive predictive value (PPV), negative predictive value (NPV), and accuracy at this threshold were reported.

#### 2.1.5. Statistical Analysis

In this study, ArcGIS software was used for geographic mapping, and R‐4.4.1 (× 64) was used for data collation and analysis. All hypothesis tests were two‐sided tests, and the hypothesis test level *α* was 0.05; that is, all statistical test results were statistically significant with *p* < 0.05.

## 3. Results

### 3.1. Basic Information of Canines

**FIGURE 1:**
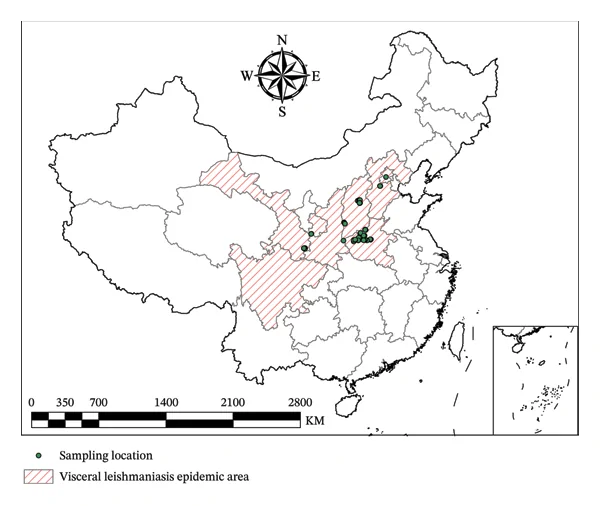
Distribution map of dog sampling points. Green dots indicate the distribution of canine sampling sites, while the red shaded area represents the endemic range of visceral leishmaniasis in mainland China.

All canine blood samples were collected from 5 provinces (Figure 1). Among them, 53 identified breeds were included clearly, while the breeds of 8 canines were uncertain. The Chinese pastoral canines were the main breed, accounting for 66.7% (Table 1). The distribution of the top 15 canine breeds among the collected canine samples is shown in Table 1.

**TABLE 1:** Distribution of major canine breeds.

Canine breeds	Number
Chinese rural dog	991 (66.78%)
Mastiff	52 (3.5%)
Teddy	51 (3.44%)
Golden Retriever	47 (3.17%)
Labrador	38 (2.56%)
Corgi	38 (2.56%)
German Shepherd	37 (2.49%)
Wolfdog	25 (1.68%)
Greyhound	23 (1.55%)
Border Collie	20 (1.35%)
Sheepdog	15 (1.01%)
Bichon Frise	13 (0.88%)
Shiba Inu	12 (0.81%)
Pomeranian	12 (0.81%)
Canis rufus	9 (0.61%)
Others	101 (6.81%)

**FIGURE 2:**
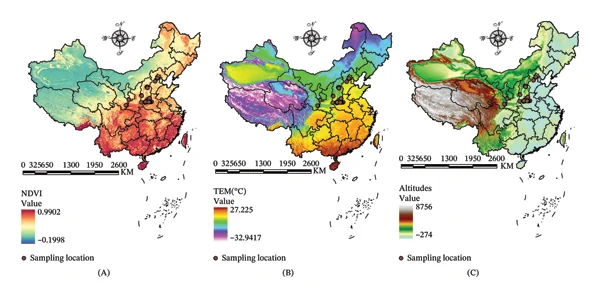
The climate and other variable conditions at the sampling point locations. (A) Mean annual temperature (°C), (B) normalized difference vegetation index (NDVI), and (C) altitude (m) across China. Red dots indicate the distribution of canine sampling points, while different colors represent the distribution patterns of various variables.

At the same time, the length of the canine’s body hair may affect the bite probability of the *Phlebotomus sinensis*. Among the 1374 canine samples collected, the gender ratio was balanced (58.2% male vs. 41.8% female), with the majority being short‐haired canines (90.4%). A univariate analysis between the negative and positive canine groups revealed that the average elevation of negative canines was 619.3 ± 475.05 m, compared to 338.3 ± 188.53 m for positive canines, and the annual average temperature of negative canines was 13.8 ± 2.57°C, while that of positive canines was 15.5 ± 0.77°C (Figure 2). These two factors showed statistically significant differences between the groups (Table 2).

**TABLE 2:** Basic information about canines.

Variable	Overall *N* = 1374^1^	Outcome grouping		*p* value^2^
		Negative *N* = 1329^1^	Positive *N* = 45^1^	
Sex				0.608
Female	799 (58.2%)	775 (58.3%)	24 (53.3%)	
Male	575 (41.8%)	554 (41.7%)	21 (46.7%)	
Canine hair length				0.348
Short‐haired canine	1242 (90.4%)	1199 (90.2%)	43 (95.6%)	
Long‐haired canine	132 (9.6%)	130 (9.8%)	2 (4.4%)	
Age (year)	3.2 ± 2.75	3.2 ± 2.75	3.4 ± 2.68	0.614
Weight (kg)	12.4 ± 10.31	12.3 ± 10.36	12.8 ± 8.67	0.741
NDVI	0.4 ± 0.08	0.4 ± 0.08	0.5 ± 0.12	0.068
Altitude	610.1 ± 471.08	619.3 ± 475.05	338.3 ± 188.53	< 0.001
Temperature (°C)	13.9 ± 2.55	13.8 ± 2.57	15.5 ± 0.77	< 0.001

^1^ *n* (%); mean ± SD.

^2^Pearson’s chi‐squared tests; Welch rwo‐sample *t*‐test.

### 3.2. Construction of Binary Logistic Regression Model

**FIGURE 3:**
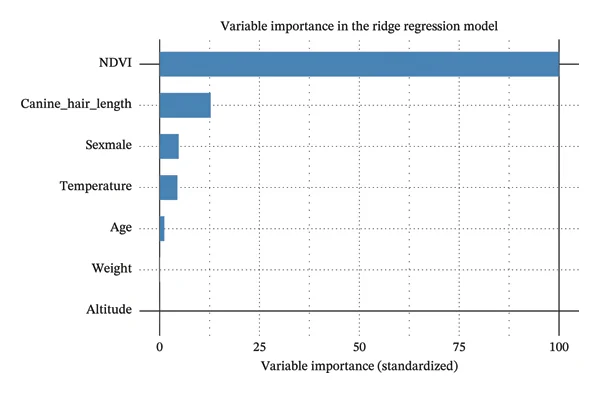
Variable importance in the ridge regression model.

The ridge regression was optimized using 10‐fold cross‐validation repeated 30 times, yielding an optimal penalty strength of *λ* = 0.0215. At this penalty level, the shrunk coefficients and odds ratios (OR) for each predictor are shown in Table 3. Among these, the NDVI had the largest absolute coefficient value (*β* = 2.675, OR = 14.51), followed by canine hair length (short vs. long) (OR = 1.41). Age, sex, and temperature exhibited only weak positive effects (OR 1.00–1.13), while the effects of weight and altitude were extremely weak. The variable importance ranking plot (Figure 3) visually illustrates this gradient: the standardized contribution of NDVI is far higher than that of other variables, with coat length and temperature ranking second and third, respectively, while the contributions of body weight and elevation are negligible. It should be noted that the extremely high OR for NDVI is amplified by its minimal variability at the raw scale; the actual change in risk per unit change remains within a reasonable range. Furthermore, all coefficients were subject to moderate ridge penalization, which avoids the extreme estimates that may occur in ordinary logistic regression and ensures the model’s stability under small sample sizes.

**TABLE 3:** Model coefficients and performance based on ridge regression‐penalized logistic regression.

Variable	*β*	OR
Canine hair length (short‐haired)	0.342	1.408
Age	0.032	1.032
Sex (male)	0.128	1.136
Weight	0.003	1.003
NDVI	2.675	14.505
Altitude	−0.001	0.999
Temperature	0.119	1.127

**FIGURE 4:**
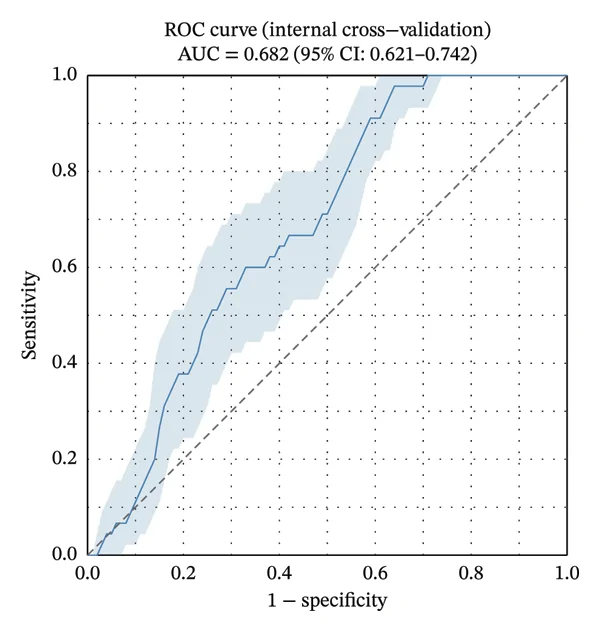
ROC curve under internal cross‐validation.

Model discriminatory power was evaluated using the same 10‐fold stratified cross‐validation with 30 repetitions. The area under the curve (AUC) of the receiver operating characteristic (ROC) curve was 0.682 (95% CI: 0.621–0.742), indicating that the model exhibits moderate discriminatory power in the current sample (Figure 4).

Based on the predicted probabilities from cross‐validation, the threshold corresponding to the maximum Youden index (0.0238) was selected as the optimal decision cutoff point (Table 4). At this threshold, the model demonstrated extremely high sensitivity (97.78%) and relatively low specificity (35.89%), meaning that the model was able to identify nearly all affected dogs, but at the cost of a high false‐positive rate. The corresponding PPV is only 4.91%, the NPV is as high as 99.79%, and the overall accuracy is 37.92%. These results are consistent with the fundamental principles of rare disease prediction: in a sample with a positive rate of only about 3.3%, high sensitivity inevitably leads to a large number of negative individuals being misclassified as positive, thereby lowering the PPV and accuracy; conversely, a high NPV indicates that individuals classified as negative by the model are highly likely to be disease‐free, making the model suitable as an auxiliary tool for exclusionary diagnosis. When the threshold is raised, specificity improves significantly (e.g., specificity rises to approximately 89% at a threshold of 0.05), but this comes at the expense of sensitivity; therefore, its use requires balancing the tolerance for missed diagnoses and false positives based on the specific clinical context. The above metrics are based on the average probabilities from internal cross‐validation and reflect the model’s expected classification performance on new samples; however, due to the small number of positive events (45 cases), the confidence intervals for the estimated sensitivity and PPV are relatively wide.

**TABLE 4:** Model performance indicator status by threshold.

Threshold	Sensitivity (%)	Specificity (%)	PPV (%)	NPV (%)	Accuracy (%)
0.0238^∗^	97.78	35.89	4.91	99.79	37.92
0.02	97.78	32.51	4.68	99.77	34.64
0.03	80.00	44.85	4.68	98.51	46.00
0.05	35.56	82.47	6.43	97.42	80.93

^∗^The threshold (0.0238) corresponding to the maximum value of the Youden index is used as the optimal decision cutoff point.

Considering the combination of discriminatory power, classification performance, and variable importance distribution, this penalized regression model provides a certain level of risk stratification capability with the current set of variables. It is particularly effective at ruling out nonaffected dogs (with an extremely high NPV), but its PPV is insufficient, and the number of false positives in individual predictions is too high, making it difficult to use directly for diagnosis. NDVI, as the factor with the highest contribution, suggests that it may be an important environmental or ecological indicator for the occurrence of this disease; however, given that its effect is amplified by the unit of measurement and there is a lack of external validation, its actual predictive weight still needs to be reevaluated in a larger sample. Overall, this model is best suited as a preliminary screening tool in scenarios with low tolerance for missed diagnoses, rather than as an independent diagnostic method. In the future, incorporating stronger predictive factors and expanding the positive sample size is expected to substantially improve the model’s discriminatory power and PPV.

## 4. Discussion

VL as a major parasitic disease in China has received attention from the state and has been provided with special funds [14]. It is precisely because of the government’s support and the efforts of professional technicians that the VL epidemic has been brought under control [15]. However, the incidence of MT‐ZVL has suddenly increased after a period of stability [16]. Therefore, it is necessary to understand the reasons for the recent increase in the incidence rate. As we all know, MT‐ZVL mainly occurs in seven provinces, including Gansu, Beijing, Henan, Hebei, and Shanxi provinces [17]. Therefore, this study aimed to determine the infection rate of canines, which are important hosts of VL, and the environmental and social factors influencing the infection rate of canines.

The average altitude of negative canines (619.3 ± 475.05 m) is higher than that of positive canines (338.3 ± 188.53 m). This finding shows that we should pay more attention to the control of sandflies in low‐altitude areas, because their transmission potential is more dangerous than that in high‐altitude areas. Published reports have found that people in Brazil who are prone to leishmaniasis live at an altitude of 780 to 880 m [18]. In Italy, researchers discovered Leishmania in horses. The study also found that altitude was significantly correlated with positive serum levels in horses, and most of the positive horses were raised in stables at an altitude of 200 to 500 m [19]. Four cross‐sectional seropositivity surveys were conducted throughout the Mediterranean region from 2012 to 2016. It was found that an important factor for the overall seropositivity was altitude [OR = 0.02 (95% CI: 0.001–0.19); *p* = 0.001], and thus altitude was identified as a risk factor for infection [20]. Serological tests were conducted on 800 canines in Murcia, southeastern Spain. The results showed that in rural areas at an altitude of 300–500 m and with an average temperature of 18.6°C–19°C from March to July, the risk of canine leishmaniosis increased significantly [21]. Greek researchers found that there s a significant correlation between serum positivity at low altitudes and in surface water [22]. The annual average temperature of negative dogs was 13.8 ± 2.57°C, while that of positive dogs was 15.5 ± 0.77°C. This indicates that the movement of sandflies in areas with a high mean annual temperature has a greater probability of transmitting VL than in areas with a low mean annual temperature. For every 100 m increase in altitude, the temperature drops by 0.6°C [23]. This also echoes the results of descriptive statistics obtained from this study.

This finding suggests that ecological factors may play a more dominant role in shaping transmission patterns in MT‐ZVL endemic areas. The vegetation index has been reported in other vector‐borne diseases, where environmental suitability often outweighs population density in determining vector presence [24, 25].

NDVI is widely used as an ecological indicator reflecting vegetation density and environmental humidity [26]. In this study, the result of NDVI showed the strongest effect among all variables (OR = 14.51), suggesting that vegetation‐related ecological conditions may play a dominant role in shaping transmission risk in MT‐ZVL endemic areas. Higher NDVI values usually correspond to more suitable habitats for sandflies, as dense vegetation can provide breeding sites, resting environments, and stable microclimatic conditions [27, 28]. Previous studies have shown that sandfly density is significantly associated with vegetation cover, particularly in rural and mountainous areas where organic matter accumulation and soil humidity are favorable for larval development[27]. Studies conducted in endemic regions of Brazil and northern Italy have similarly reported that areas with higher NDVI values were associated with increased risk of *Leishmania* transmission [29, 30]. Therefore, the strong association observed in this study further supports the role of NDVI in sustaining transmission cycles of MT‐ZVL. An interesting finding of the present study was that coat length was also associated with canine *Leishmania* infection. To our knowledge, few studies have evaluated the potential influence of canine coat characteristics on the risk of *Leishmania* infection in China. Although the underlying mechanism remains unclear, it is possible that coat length may influence the accessibility of sandflies to preferred biting sites, or alternatively reflect differences in dog management practices, living environments, or host characteristics that are associated with vector exposure. Therefore, the observed association should be interpreted cautiously and does not imply a causal relationship. Further epidemiological and entomological studies are warranted to investigate the biological mechanisms underlying this association and to determine whether coat characteristics could serve as a useful indicator for identifying dogs at increased risk of infection.

In contrast, the OR < 1 for altitude. The cumulative increases in elevation may still result in substantial reductions in infection risk. This finding is consistent with the ecological characteristics of sandflies, which are highly sensitive to temperature and atmospheric conditions [23]. Nevertheless, given that the OR was close to 1, more evidence is needed to substantiate this association. At higher altitudes, lower temperatures and environmental constraints may limit sandfly activity, survival, and breeding capacity [31, 32]. Additionally, human and canine population densities tend to be lower in high‐altitude areas, which may further reduce transmission opportunities. Interestingly, this study found that individual‐level characteristics of canines, including sex, age, weight, and hair length, were not significantly associated with infection status. NDVI factors may play a more dominant role than host biological characteristics in determining infection risk. Similar findings have been reported in previous studies, where canine infection was more strongly associated with environmental exposure rather than intrinsic host factors [33]. However, behavioral variables such as outdoor activity or sleeping location, which are known to influence exposure risk, were not included in this study and may explain the lack of association. In terms of model performance, this model has a sensitivity of 97.78%, indicating that the selected variables can effectively screen out positive cases. The model can effectively distinguish dog samples in high‐risk areas and can carry out preventive treatment for dogs in these areas. Therefore, future studies should incorporate larger and more balanced datasets to improve model generalizability.

This study has several important public health implications. First, it highlights that the environmental factor NDVI is a key determinant of canine infection and should be integrated into surveillance and early warning systems. Secondly, the positive rate of canine infection is an important reservoir and an early indicator of transmission risk. Therefore, it is of great public health significance to distinguish dogs in high‐risk areas. Targeted interventions, such as vector control and management of infected dogs, should be prioritized in high‐risk ecological settings.

Nevertheless, several limitations should be acknowledged. First, this study used cross‐sectional data, which limits causal inference. Second, some key variables, such as sandfly density and canine behavior, were not included. Third, the spatial resolution of environmental data may not capture microhabitat variability. Finally, external validation in other endemic regions is needed.

In conclusion, this study provides new evidence that environmental factors, particularly NDVI, are significantly associated with canine infection in MT‐ZVL endemic areas in China, offering important insights for targeted control strategies.

## Author Contributions

Dr. Shizhu Li and Zhongqiu Li designed the study. Zhengbin Zhou, Zhongqiu Li, and Shuxun Wang collected the samples. Zhongqiu Li and Haobo Ni analyzed the data and prepared draft figures. Zhongqiu Li and Zixin Wei prepared the manuscript draft with important intellectual input from Dr Junhu Chen and Yi Zhang.

## Funding

The study was supported by the National Natural Science Foundation of China (No. 82473688) as well as the Prevention and Control of Emerging and Major Infectious Diseases‐National Science and Technology Major Project (2025ZD01901100 and 2025ZD01901104).

## Disclosure

All authors approved the final manuscript. All authors had complete access to the study data.

## Ethics Statement

The medical Ethics Committee of the National Institute of Parasitic Diseases (NIPD), Chinese Center for Disease Control and Prevention (Chinese Center for Tropical Diseases Research), approved this study (2021019).

## Consent

The authors have nothing to report.

## Conflicts of Interest

The authors declare no conflicts of interest.

## Data Availability

The data supporting the findings of this study are available from the corresponding author, Shizhu Li, upon reasonable request.
